# Effects of Estradiol and Methoxychlor on Leydig Cell Regeneration in the Adult Rat Testis

**DOI:** 10.3390/ijms15057812

**Published:** 2014-05-06

**Authors:** Bingbing Chen, Dongxin Chen, Zheli Jiang, Jingyang Li, Shiwen Liu, Yaoyao Dong, Wenwen Yao, Benson Akingbemi, Renshan Ge, Xiaokun Li

**Affiliations:** 1 Department of Pharmacy, School of Pharmacy of Wenzhou Medical University, Chashan District, Wenzhou 325000, Zhejiang, China; E-Mails: bb55332@163.com (B.C.); cdx19891029@sina.com (D.C.); jiang zheli@163.com (Z.J.); 15258693527@163.com (Y.D.); 15258672295@163.com (W.Y.); 2 Research Academy of Reproductive Biomedicine, the 2nd Affiliated Hospital, Wenzhou Medical University, Wenzhou 325027, Zhejiang, China; E-Mails: jingyanglee0928@163.com (J.L.); 18367812261@163.com (S.L.); raygee0828@163.com (R.G.); 3 Department of Anatomy, Physiology and Pharmacology, Auburn University, Auburn, AL 36948, USA; E-Mail: akingbt@vetmed.auburn.edu

**Keywords:** Leydig cells, steroidogenesis, regeneration, methoxychlor, estrogen

## Abstract

The objective of the present study is to determine whether methoxychlor (MXC) exposure in adulthood affects rat Leydig cell regeneration and to compare its effects with estradiol (E2). Adult 90-day-old male Sprague-Dawley rats received ethane dimethane sulfonate (EDS) to eliminate the adult Leydig cell population. Subsequently, rats were randomly assigned to four groups and gavaged with corn oil (control), 0.25 mg/kg E2 and 10 or 100 mg/kg MXC daily from days 5 to 30 post-EDS treatment. The results showed that MXC and E2 reduced serum testosterone levels on day 58 post-EDS treatment. qPCR showed *Hsd17b3* mRNA levels were downregulated 7–15 fold by E2 and MXC, indicating that development of the new population of Leydig cells was arrested at the earlier stage. This observation was supported by the results of histochemical staining, which demonstrated that Leydig cells in MXC-treated testis on day 58 post-EDS treatment were mostly progenitor Leydig cells. However, *Pdgfb* mRNA levels were downregulated, while *Lif* transcript levels were increased by MXC. In contrast, E2 did not affect gene expression for these growth factors. In conclusion, our findings indicated that both MXC and E2 delayed rat Leydig cell regeneration in the EDS-treated model, presumably acting by different mechanisms.

## 1. Introduction

Methoxychlor (MXC) is an organochlorine insecticide that is widely utilized in the agriculture sector [1–4]. MXC had been considered a good replacement of a similar organochlorine pesticide, dichlorodiphenyl trichloroethane (DDT), which is more toxic and has a long half-life (about eight years) and accumulates in body tissues [4]. Unlike DDT, MXC has a shorter half-life. In mouse, when 50 mg/kg of radiolabeled methoxychlor was orally administered, almost all the radioactivity was excreted in feces and urine within 48 h [4]. Previous observations suggest that MXC is a reproductive toxicant, albeit with weak estrogenic activity [3], and its bioactive metabolite, 2,2-bis(p-hydroxyphenyl)-1,1,1-trichloroethane (HPTE), caused greater endocrine-disrupting toxicity in both sexes than the parent MXC compound [1,5]. For example, both MXC and HPTE inhibited androgen production by rat Leydig cells [1,5]. Furthermore, MXC and HPTE suppressed steroidogenic enzyme activities in both human and rat testes *in vitro*, including 3β-hydroxysteroid dehydrogenase (3β-HSD), 17β-hydroxysteroid dehydrogenase 3 (17β-HSD3) and 11β-hydroxysteroid dehydrogenases (11β-HSD) [5,6], which presumably contribute to their inhibitory effects on androgen secretion.

Several studies have shown that estradiol blocks the regeneration of the Leydig cell population in rats after treatment with the Leydig cell cytotoxin, ethane dimethane sulfonate (EDS) [7]. EDS is an alkylating agent, which specifically causes the elimination of Leydig cells [8] within seven days of administration. However, mesenchymal stem cells (MSCs) are able to develop and differentiate a new population of Leydig cells in the adult testis. The increase in the population of newly-regenerated Leydig cells in the following eight weeks in the rat testis is stimulated by high circulatory levels of pituitary luteinizing hormone [9]. This pattern of development recapitulates the process of Leydig cell development in the growing rat. For example, MSC-derived progenitor Leydig cells gradually differentiate into immature Leydig cells from days 21 to 28 and, then, into mature adult Leydig cells on day 56 day post-EDS treatment [8,10]. In the middle of the regeneration, 5α-androstane-3α,17β-diol (DIOL) is the primary androgen, which is produced by immature Leydig cells, due to the high expression of 5α-reductase 1 and 3α-hydroxysteroid dehydrogenase [8,10]. It was observed that the E2 effects on rat Leydig cell regeneration depends on the time of exposure to this agent [7]. Exposure to E2 during the initial five days post-EDS treatment did not affect Leydig cell regeneration. However, exposure to E2 during the 5–30 days period after EDS treatment blocked Leydig cell division [7]. MXC has been shown to have weak estrogenic activity [3]. However, whether it has similar action to estrogen in the inhibition of Leydig cell regeneration is unclear. In the present study, we compared MXC and E2 effects on the capacity for Leydig cell regeneration in the EDS-depleted testis. The results can provide a research basis for sexual dysfunction caused by pollutants.

## 2. Results

### 2.1. General Reproductive Toxicology

Gavage of rats with 0.25 mg/kg/day E2 or 10 or 100 mg/kg/day MXC from day 5 to 30 post-EDS did not affect body weights, except in the group of 10 mg/kg MXC, which significantly increased rat body weights 14 days post-EDS (Table 1). In all groups, rats did not show any signs of distress.

### 2.2. Serum Testosterone and DIOL Levels

**Figure 1.**
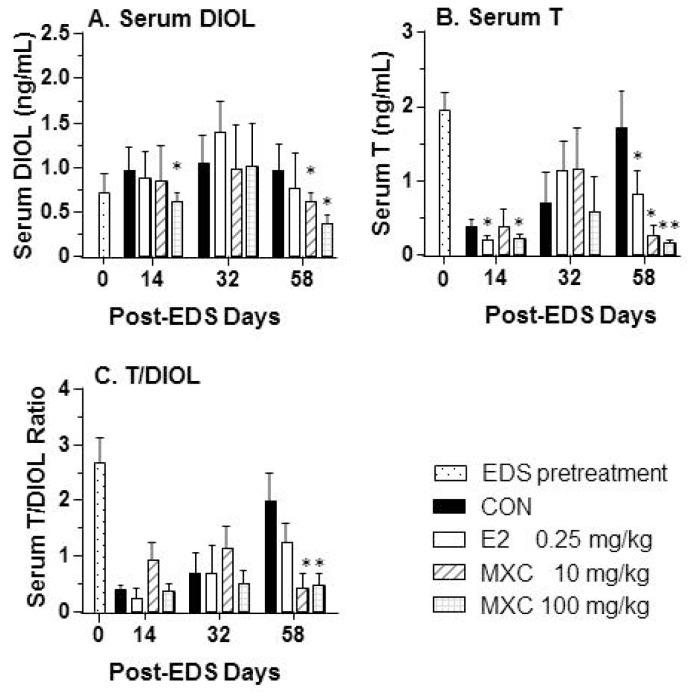
Serum testosterone (T) and 5α-androstane-3α,17β-diol (DIOL) levels after treatment of estradiol (E2) or methoxychlor (MXC) from days 5 to 30 post-EDS. (**A**) DIOL; (**B**) T; (**C**) T/DIOL ratio. Mean ± SEM, *n* = 5. *, ** indicate significant difference when compared to control (CON) at each time point at *p* < 0.05 and 0.01, respectively.

In the control group, 7 days after testicular artery injection of 10 mg/kg of EDS, serum testosterone levels were undetectable (data not shown), indicating that Leydig cells were completely eliminated. Serum testosterone concentrations in adult Sprague-Dawley male rats not treated with EDS were determined to be about 2 ng/mL (Figure 1). As shown in Figure 1A, serum testosterone or DIOL concentrations were significantly decreased by 100 mg/kg MXC on days 14 and 58 post-EDS. E2 also decreased testosterone, but not DIOL levels on days 14 and 58 post-EDS. We measured DIOL (Figure 1A) and testosterone concentrations (Figure 1B) and determined testosterone/DIOL ratios in order to track Leydig cell regeneration. The effects of MXC and E2 on DIOL concentrations showed a similar trend as testosterone plus DIOL concentrations. Furthermore, E2 suppressed testosterone production on days 14 and 58 post-EDS treatment. Testosterone/DIOL ratios on day 14 post-EDS treatment in control animals were 0.402 ± 0.040 (Figure 1C), indicating that Leydig cells were progenitors/immature at this stage. The serum testosterone level in the control rat on day 58 post-EDS treatment was around 2 ng/mL, implying that Leydig cells are fully regenerated. However, on day 58 post-EDS treatment, both 10 and 100 mg/kg of MXC decreased testosterone/DIOL ratios to 0.418 ± 0.124 and 0.466 ± 0.108, which was similar to control levels on day 14 post-EDS treatment. This finding indicates that MXC delayed the process of Leydig cell differentiation. However, E2 did not affect testosterone/DIOL ratios on day 58 post-EDS treatment, indicating that the Leydig cell maturation was unaffected by E2.

### 2.3. Effects of E2 and MXC on Leydig Cell Number and Maturity, as well as Spermatogenesis

**Figure 2.**
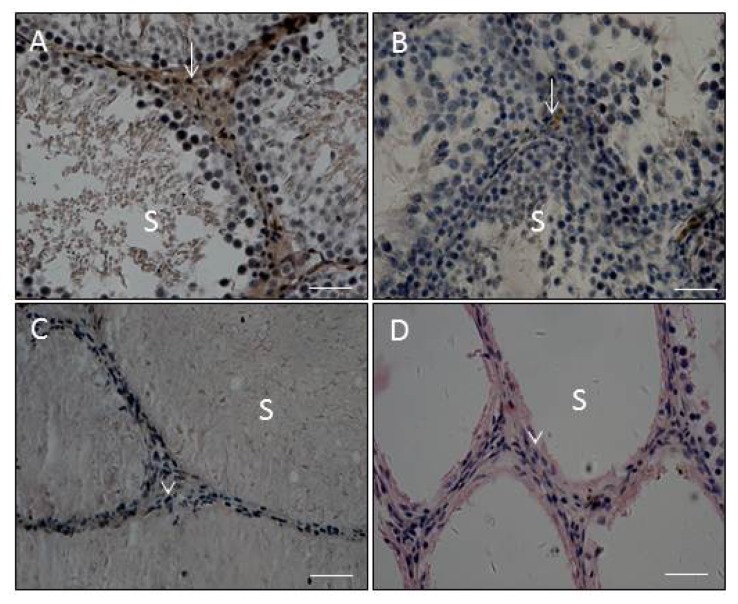
Immunohistochemical staining of 58-day-post-EDS rat testis sections from estradiol (E2) or methoxychlor (MXC). (**A**) Control group; (**B**) E2 group; (**C**) 10 mg/kg of MXC group; (**D**) 100 mg/kg of MXC group. Brown cytosolic staining was 11β-hydroxysteroid dehydrogenase 1, the biomarker of more mature Leydig cells. White arrows point to the Leydig cells. White arrowheads point to the unstaining spindle-shaped cell. S, seminiferous tubules. Bar = 50 μm.

**Figure 3.**
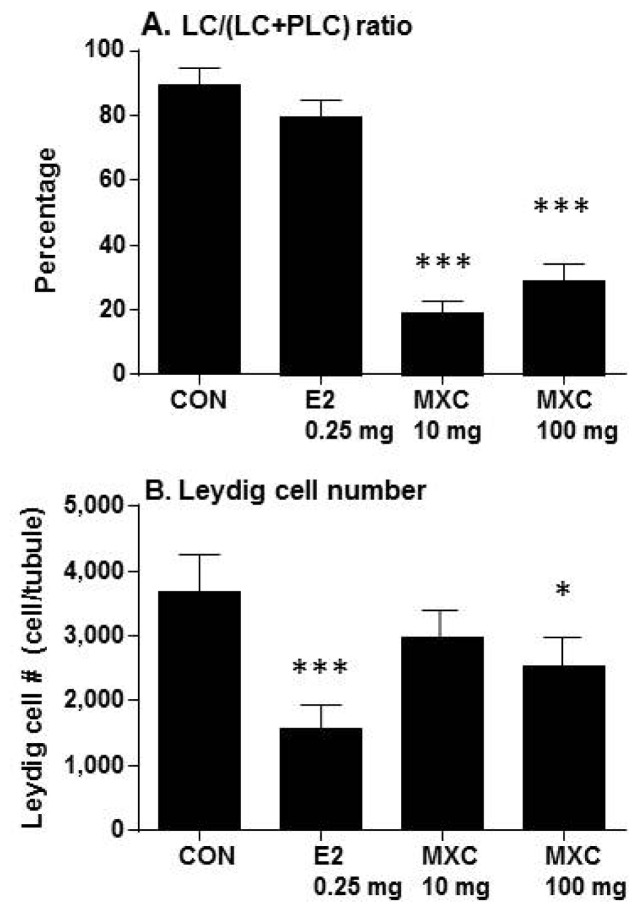
Leydig cell numbers and the ratio of mature Leydig cell to progenitor Leydig cell in 58-day-post-EDS rat testis after estradiol (E2) or methoxychlor (MXC) treatment. (**A**) Leydig cell to the total of progenitor and leydig cell ratio; (**B**) Leydig cell number. Mean ± SEM, *n* = 5. *, *** indicate significant difference when compared to the control (CON) at *p* < 0.05 and 0.001, respectively.

As reported in the previous studies, 14 and 32 days post-EDS, there are progenitor and immature Leydig cells in the control testis, respectively [11,12]. Therefore, the comparison of maturation in these earlier time points was difficult. Fifty eight days post-EDS, Leydig cells were all mature [11,12]; therefore, testis sections were evaluated on day 58 post-EDS treatment. After 10 or 100 mg/kg of MXC treatment, on the day 58 post-EDS testis section, Leydig cells exhibited spindle-shaped nuclei and no 11β-HSD1 staining, indicating that these Leydig cells are mesenchymal progenitor cells (Figure 2D). The ratio of round cells (more mature Leydig cells) to spindle-shaped cells (progenitor cells) was significantly reduced (Figure 3A). However, E2 did not affect the ratio of round cells to spindle-shaped cells (Figure 3A), and 11β-HSD1 positive cells were detected in E2-exposed testis (Figure 2B). Since the E2 and MXC did not alter the diameters of seminiferous tubules (data not shown), the number of Leydig cells, including progenitor, immature and adult Leydig cells, were counted and adjusted as the Leydig cell number per tubule. However, E2 significantly decreased Leydig cell numbers in the testis on day 58 post-EDS (Figure 3B). In contrast, the total number of Leydig cells in the MXC-treated (10 mg/kg) testes were not altered (Figure 3B), indicating that this dose of MXC did not affect Leydig cell proliferation, whereas the higher dose of MXC (100 mg/kg) slightly, but significantly reduced Leydig cell numbers. In the control and E2-treated testes, there were some spermatocytes and round spermatids at day 58, suggesting that spermatogenesis is recovering (Figure 2A,B). However, the recovery of spermatogenesis in the MXC-treated testis was delayed. Especially in 100 mg/kg of MXC-treated testis, there were many seminiferous tubules that were empty of spermatocytes and round spermatids (Figure 2D).

### 2.4. Effects of E2 and MXC on Genes Related to Leydig Cell Regeneration

**Figure 4.**
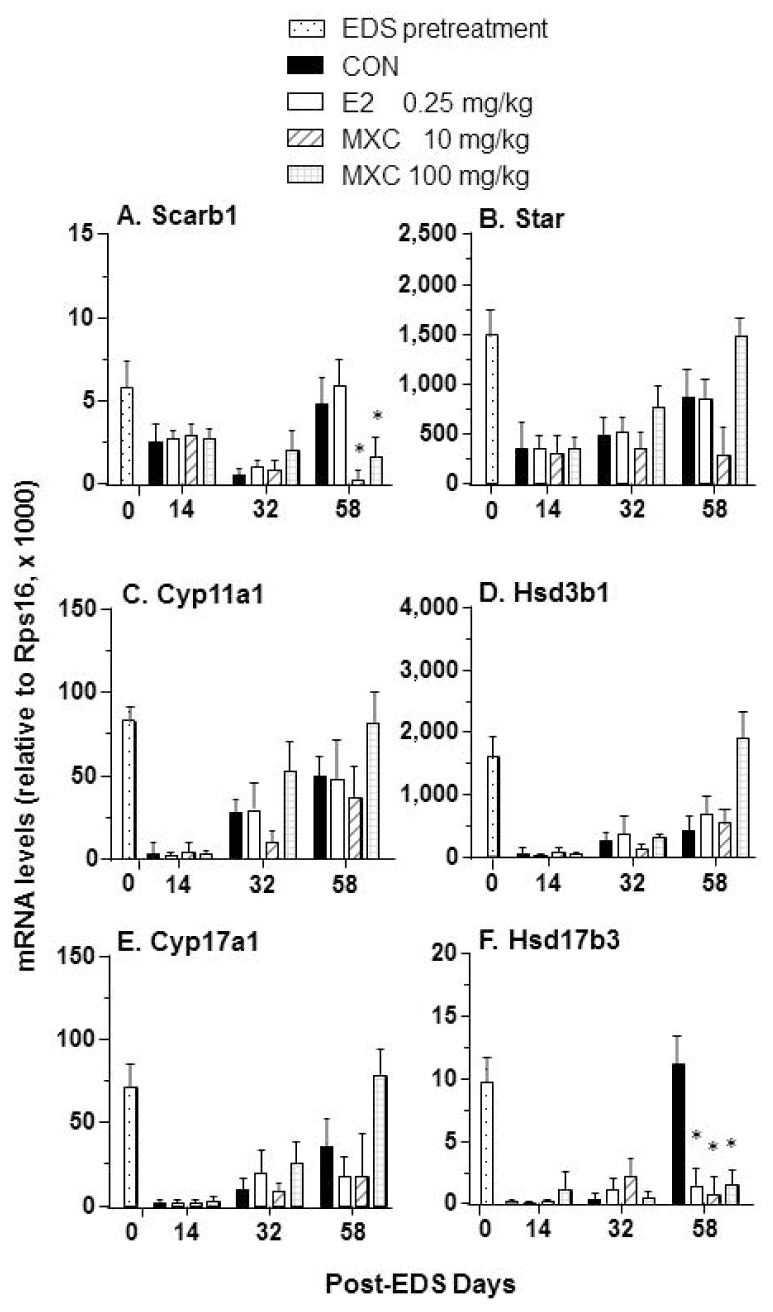
Quantitative PCR assay of the Leydig cell differentiation-related mRNA levels. (**A**) Scarb1; (**B**) Star; (**C**) Cyp11a1; (**D**) Hsd3b1; (**E**) Cyp17a1; (**F**) Hsd17b3. Mean ± SEM, *n* = 5. * indicates significant difference when compared to the control (CON) at each time point at *p* < 0.05.

Transcript levels for 12 testis-specific genes were examined by qPCR to assess the effects of E2 and MXC on Leydig cell differentiation during the regeneration process. Exposure to 10 and 100 mg/kg of MXC after EDS treatment significantly decreased the expression of *Scarb1* and *Hsd17b3* on day 58 post-EDS treatment, whereas E2 reduced *Hsd17b3* expression on day 58 post-EDS treatment (Figure 4).

**Figure 5.**
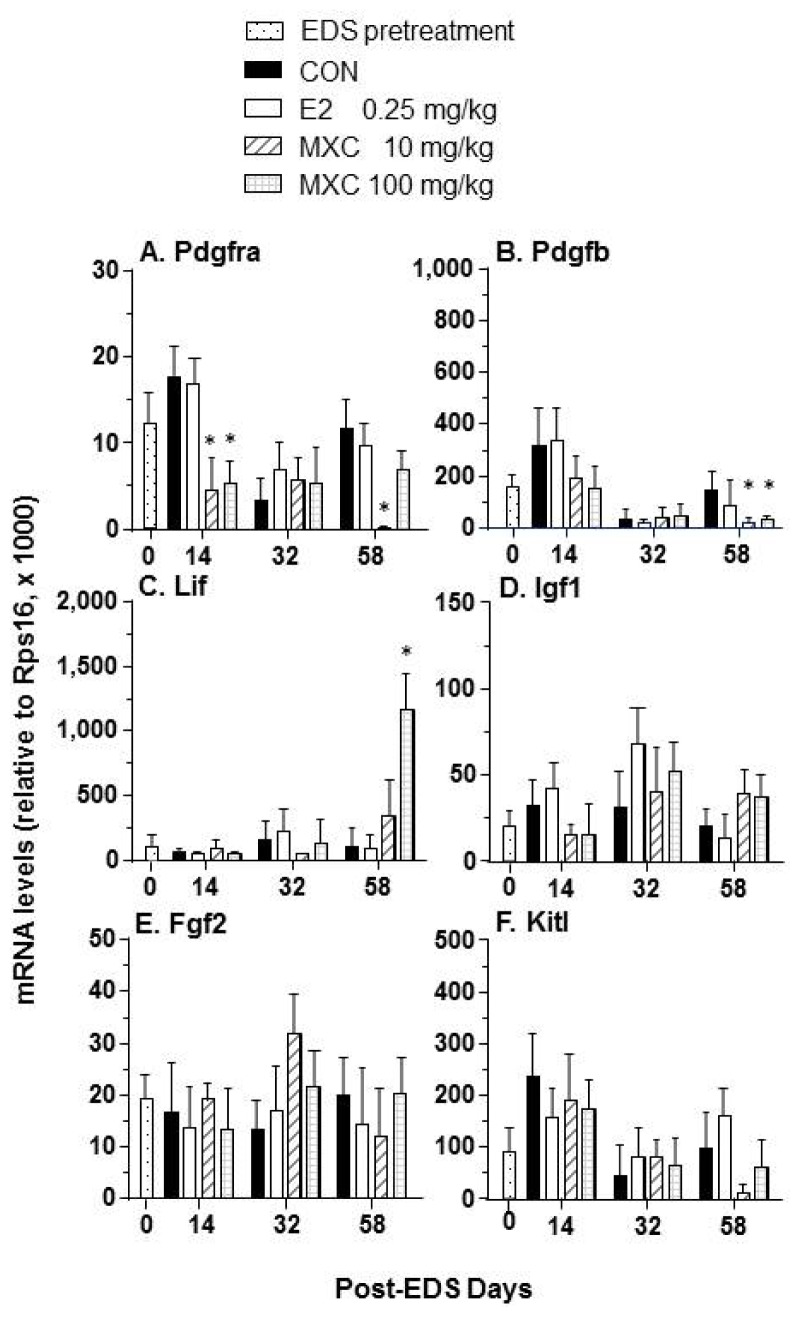
Quantitative PCR assay of the growth factor/receptor gene expression levels of the testis. (**A**) *Pdgfra*; (**B**) *Pdgfb*; (**C**) *Lif*; (**D**) *Igf1*; (**E**) *Fgf2*; (**F**) *Kitl*. Mean ± SEM, *n* = 5. * indicates significant difference when compared to the control (CON) at each time point at *p* < 0.05.

Our previous study indicates that several growth factors and their cognate receptors are involved in the regulation of Leydig cell differentiation [13]. As shown in Figure 5, *Pdgfb* expression levels were decreased in all MXC-treated testes on day 58 post-EDS treatment. The levels of *Pdgfra* mRNA were downregulated by 10 and 100 mg/kg of MXC on day 14 post-EDS treatment and by the 10 mg/kg of MXC dose at 58 days post-EDS treatment (Figure 5A). In contrast, the *Lif* expression level was increased after exposure to 100 mg/kg MXC by day 58 post-EDS treatment (Figure 5C). However, E2 had no effects on the expression of these growth factor, as well as *Pdgfra* expression levels.

### 2.5. Androgen Biosynthetic Enzyme Activities

**Figure 6.**
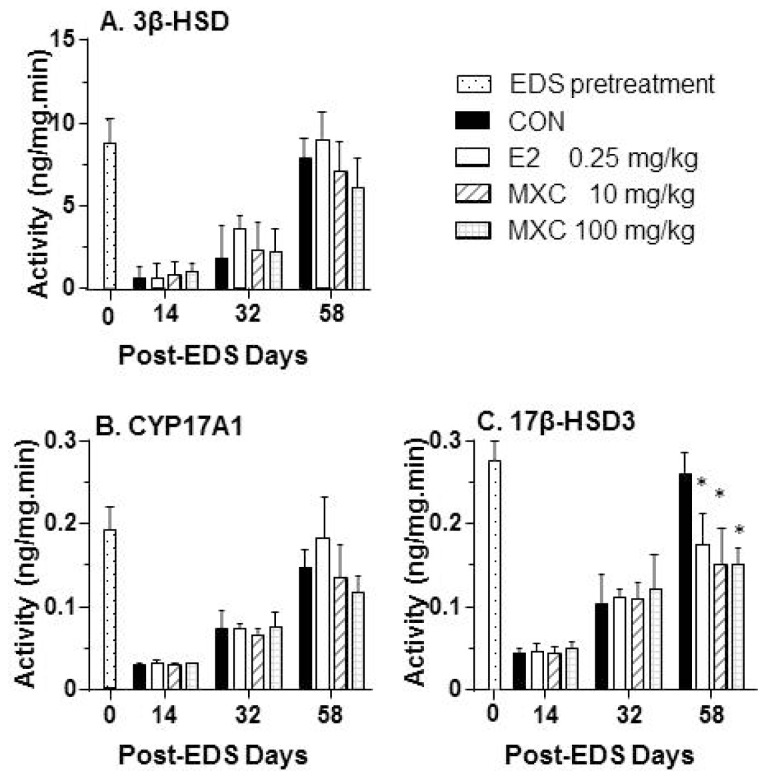
Steroidogenic enzyme activities of testis after estradiol (E2) and methoxychlor (MXC). (**A**) 3β-HSD; (**B**) CYP17A1; (**C**) 17β-HSD3. Mean ± SEM, *n* = 5. * indicates significant difference when compared to the control (CON) at each time point at *p* < 0.05.

Evaluation of androgen biosynthetic enzyme protein levels showed that 3β-HSD activities were not affected by the administration of either the E2 or MXC treatment to EDS-treated rats (Figure 6) in tandem with mRNA levels. In contrast, 17β-HSD3 activities were decreased after exposure of EDS-treated rats to E2 and MXC, also in parallel with *Hsd17b3* mRNA levels (Figure 6). Cyp17A1 was not affected by the exposure of EDS-treated rats to MXC and E2.

### 2.6. Effects of E2 and MXC on Genes in the Pituitary

**Figure 7.**
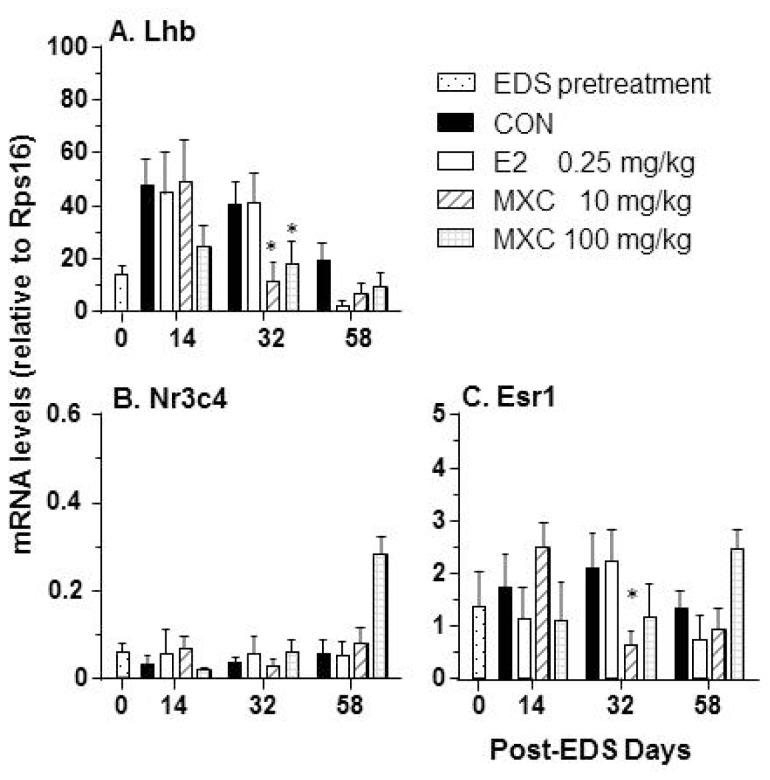
Quantitative PCR assay of mRNA levels in the pituitary. (**A**) *Lhb*; (**B**) *Nr3C4*; (**C**) *Esr1*. Mean ± SEM, *n* = 5. * indicates significant difference when compared to the control (CON) at each time point at *p* < 0.05.

As described in Figure 7, both doses of MXC suppressed *Lhb* expression on day 32 post-EDS treatment. However, this suppression was alleviated after termination of MXC exposure after day 30 post-EDS treatment. We also observed that exposure to 10 mg/kg of MXC suppressed expression of *Esr1* in the pituitary gland on day 32 post EDS treatment.

## 3. Discussion

The EDS-treated rat model is very unique for studying the differentiation of Leydig cells in the adult testis, because Leydig cells in the adult testis are completely eliminated after a single intraperitoneal injection of EDS (75 mg/kg) [8] or a single intra-testicular artery injection of 2.5 mg/testis of EDS (in the present study). The loss of Leydig cells leads to the elevation of circulating luteinizing hormone (LH), which, together with local growth factors or cytokines, promotes the proliferation of Leydig precursor cells and their differentiation. The present study demonstrated that the exposure of adult rats to MXC during the critical window of days 5 to 30 post-EDS treatment resulted in the delayed maturation of Leydig cells, as assessed on day 58 post-EDS treatment.

It appears that testis weight in the post-EDS treatment period does not adequately reflect Leydig cell function, because the testis weights were decreased after EDS treatment associated with decreased spermatogenesis and diminished testosterone stimulation [8,11,12]. Interestingly, exposure to both low (10 mg/kg) and high doses (100 mg/kg) of MXC suppressed the differentiation of Leydig cells, as indicated by the decreased expression of *Scarb1* and *Hsd17b3* and testosterone levels. The levels of testicular *Hsd17b3* mRNA in rats exposed to E2 and MXC on day 58 post-EDS treatment were similar to the control levels 32 days after EDS treatment, indicating that most Leydig cells remained at the progenitor or immature stages of development. These observations were supported by histochemical studies showing that interstitial cells in MXC-exposed testis on day 58 post-EDS were mostly spindle-shaped without 11β-HSD1 staining, which is a biomarker for Leydig cells beyond progenitor Leydig cells, although Leydig cell numbers were not different from the control EDS group. The deficits in Leydig cell development resulted in decreased testosterone production.

The results demonstrated that E2 exerted a window-specific effect on Leydig cell regeneration in the EDS-treated rat model [14] during the period between days 5 to 30 post-EDS treatment [14]. At this time, E2 primarily suppressed the proliferation of Leydig cell progenitors, which led to a decrease in the number of Leydig cells [14]. This finding is in alignment with the previous observation by Abney and Myers [7]. In contrast, MXC seems to act by a different mechanism to delay Leydig cell maturation. After treatment with both 10 and 100 mg/kg MXC, Leydig cells on day 58 post-EDS testes were at the progenitor Leydig cell stage with a very lower expression of levels of the *Hsd17b3* enzyme. However, the Leydig cells in the E2-treated testis were round and also had a very lower expression of levels of *Hsd17b3*, indicating that these round cells are immature Leydig cells. Therefore, MXC induced the suppression of Leydig cell maturation at the earlier stage, while E2 did that at the later stage (immature stage). Although there was a difference for the toxicokinetic parameters between MXC (*t*_1/2_ = 22.54 ± 6.31 h) [15] and E2 (*t*_1/2_ about 10 h) [16], this difference cannot completely explain the different effects between MXC and E2.

The mechanism of MXC-induced suppression of Leydig cell maturation in the EDS-treated rat model is possibly related to the disruption of growth factor function. Normally, *Pdfgra* is exclusively expressed in Leydig cells with expression levels increasing during the process of Leydig cell development [17]. It happens that the knockout of *Pdgfa* (corresponding to the expression of the PDGF-A subunit) had the effect of delaying Leydig cell differentiation [18]. Exposure to 10 and 100 mg/kg of MXC was found to downregulate *Pdgfra* and *Pdgfb* expression [19].

Inhibition of Leydig cell differentiation by MXC may be related at least in part to the suppression of LH stimulated function. Both doses of MXC downregulated pituitary *Lhb* expression, the biologically active subunit of LH. This suppression occurred on day 32 post-EDS treatment. LH is critically required for the differentiation of Leydig cells at the later stages, and LH receptor mutant mice rendered Leydig cells infantile [20]. MXC also downregulated *Esr1* expression in the pituitary gland, thereby disrupting estrogen receptor 1-mediated signaling, as was previously described for the industrial chemical, bisphenol A [21]. Surprisingly, we did not find that E2 inhibited *Lhb* and *Esr1* expression levels, indicating that this dose of E2 may not interfere with pituitary glands. Therefore, the effects of E2 on Leydig cell proliferation possibly took place in the testis locally.

## 4. Experimental Section

### 4.1. Materials

Methoxychlor and E2 were purchased from Sigma (Sigma, St. Louis, MO, USA). An avidin-biotin immunostaining kit (PK-6101) was purchased from Vector Laboratories, Inc. (Burlingame, CA, USA). Progesterone, androstenedione, 5α-androstane-3α,17β-diol (DIOL) and testosterone radioimmunoassay kit, 11β-hydroxysteroid dehydrogenase 1 rabbit polyclonal antibody, as well as EDS were purchased from Pterosaur Biotech Co. (Hangzhou, China). The protein concentration determination kit (No. 500-0006) was purchased from Bio-Rad Laboratories, Inc. (Hercules, CA, USA).

### 4.2. Animal Treatment

EDS was dissolved in a mixture of dimethyl sulfoxide (DMSO) and water (DMSO: H2O, 1:4, *v*/*v*) for administration into the testicular artery. Sixty 90 day-old male Sprague-Dawley rats (Animal Center, Wenzhou Medical University, Wenzhou, China) received a single testicular artery injection of EDS to eliminate Leydig cells. Rats were anesthetized with a mixture of halothane/oxygen. Testes were partially delivered through separate incisions over the left and right scrotum. EDS solution (2.5 mg/testis) was prepared in sterile water: DMSO mixture and injected via the testicular artery gently. A total volume of 25 μL was injected per testis via a 30-gauge needle fitted to a 100-μL Hamilton syringe. Rats were then randomly divided into four groups: vehicle, 0.25 mg/kg/day of E2 and 10 or 100 mg/kg/day of MXC (15 animals per group). Rats were gavaged with chemicals in corn oil from 5 to 30 days post-EDS treatment (0.5 mL/250 g rat). The dosage of E2 was selected based on a previous study, in which the gavage of E2 (0.25 mg/kg/day) inhibited Leydig cell regeneration in the EDS-treated mature rat [14]. The dosage of MXC was selected based on a previous study, in which the lowest-observed-effect level for MXC inhibition of testosterone production in the rat was calculated to be 25 mg/kg [22], whereas the binding potency of HPTE to estrogen receptor-α was 17 fold less than that for E2 [23]. At the end of the treatment period, five rats from each group were sacrificed at days 14, 32 and 58 post-EDS treatment by asphyxiation with CO_2_. Trunk blood was collected, placed in a gel glass tube and centrifuged at 1500× *g* for 10 min to collect serum. Serum samples were stored at −20 °C until analyzed for testosterone by Radio Immunoassay (RIA). Furthermore, one testis from each animal was removed, frozen in liquid nitrogen and stored in −80 °C for subsequent analysis of steroidogenic enzyme transcript levels. The contralateral testis from each animal was hole-punched using a needle and then fixed in Bouin’s solution for histochemical analysis. Five animals from each group were sampled for pituitary collection. Pituitary was collected and stored at −80 °C until extraction of total RNAs. All studies were approved by the Wenzhou Medical University’s Animal Care and Use Committee.

### 4.3. Determination of Leydig Cell Number

After dehydration in ethanol and xylene, five testes selected per group per each time point were embedded in paraffin. Six micrometer-thick transverse sections were prepared and mounted on glass slides. Avidin-biotin immunostaining was performed according to the manufacturer’s instructions (Vector, Burlingame, CA, USA). Antigen retrieval was carried out by microwave irradiation for 10 min in 10 mM (pH 6.0) of citrate buffer, and endogenous peroxidase was blocked with 0.5% of H_2_O_2_ in methanol for 30 min. Sections were then incubated with an 11β-HSD1 polyclonal antibody diluted 1:1000, for 1 h at room temperature. The antibody-antigen complexes were visualized with diaminobenzidine alone, resulting in brown cytoplasmic staining in positively-labeled Leydig cells at the more advanced stage. The sections were counterstained with Mayer hematoxylin, dehydrated in graded concentrations of alcohol and cover-slipped with resin (Permount, SP15-100; Fisher Scientific, Thermo Fisher Scientific, Waltham, UK). Negative control sections were incubated with non-immune rabbit IgG (*i.e*., minus 11β-HSD1) using the same working dilution as the primary antibody. The round cells in the interstitial area with 11β-HSD1 staining represent the Leydig cells (immature and adult Leydig cells), while cells with spindle-shaped nuclei represent progenitor Leydig cells. Leydig cell numbers were calculated per 100 tubules. The ratio of Leydig cells to progenitor Leydig cells was also calculated.

### 4.4. Quantitative PCR

Total RNA was extracted from the selected five testes per group per each time point processed in TRIzol according to the manufacturer’s instructions (Invitrogen, Carlsbad, CA, USA). Real-time PCR (qPCR) was performed as previously described [24]. Ribosomal protein S16 (*Rps16*) mRNA levels were assayed in all samples as the internal control. The expression levels of 12 genes, which regulate steroidogenesis [25], were analyzed, including cholesterol transport protein genes, e.g., scavenger receptor class B member 1 (*Scarb1*) and steroidogenic acute regulatory protein (*Star*); steroidogenic enzyme genes, e.g., P450 cholesterol side chain cleavage enzyme (*Cyp11a1*), 3β-hydroxysteroid dehydrogenase 1 (*Hsd3b1*), P450 17α-hydroxylase/10-lyase (*Cyp17a1*) and 17β-hydroxysteroid dehydrogenase 3 (*Hsd17b3*); growth factor membrane receptor gene platelet growth factor receptor α (*Pdgfra*); and testicular growth factors, e.g., insulin growth factor1(*Igf1*), leukemia inhibitory factor (*Lif*), kit ligand (*Kitl*), fibroblast growth factor 2 (*Fgf2*) and platelet growth factor B subunit (*Pdgfb*). Pituitary nuclear receptors and transcription factors estrogen receptor α (*Esr1*), androgen receptor (*Nr3c4*) and pituitary luteinizing hormone (*Lhb*) gene were also measured.

### 4.5. Homogenization and Protein Content Assay

Testes samples from each group (*n* = 5) and for each time point were homogenized in 1 mL of ice-cold 0.1 M PBS (pH 7.2) containing 0.25 M sucrose. Supernatants were collected by centrifugation at 700× *g* for 30 min, removing the cell debris and keeping the mitochondrial and microsomal proteins. Supernatants were used to measure the enzyme activities of CYP17A1, 3β-HSD1 and 17β-HSD3. The protein concentrations were determined using the Bio-Rad Protein Assay Kit (cat# 500-0006; Bio-Rad, Hercules, CA, USA) according to the manufacturer’s protocol.

### 4.6. Enzyme Assay

Enzymes activities for CYP17A1, 3β-HSD1 and 17β-HSD3 were determined as previously described [26]. Briefly, the reaction mixtures (total volume of 250 mL) containing 25–160 mg of protein, 0.2 mM of cofactors (NAD+ for 3β-HSD1, NADPH for CYP17A1 and 17β-HSD3) and 1000 nM of steroid substrates (radiolabeled plus cold substrates) were incubated in a shaking water bath at 34 °C for 1–2 h. The substrates were pregnenolone (for 3β-HSD1), progesterone (for CYP17A1) and androstenedione (for 17β-HSD3). Pilot assays were performed to determine the linear reaction curve using different concentrations of proteins and for different time periods. After the reactions were terminated, media were assayed for progesterone (for 3β-HSD1), androstenedione (for CYP17A1) and testosterone (for 17β-HSD3).

Because testosterone is the major androgen produced by adult rat Leydig cells and DIOL is the major androgen produced by immature Leydig cells [10,27], serum testosterone and DIOL concentrations were measured with a tritium-based radioimmunoassay, as described [28]. Inter-assay and intra-assay variations of the testosterone and DIOL were between 7% and 8%.

The assay of progesterone, androstenedione and testosterone by RIA was performed as described previously [27,28]. Inter-assay and intra-assay variations of progesterone, androstenedione and testosterone were within 15%. The progesterone, androstenedione and testosterone levels were measured for the calculation of the activities of 3β-HSD1, CYP17A1 and 17β-HSD3, respectively.

### 4.7. Statistical Analysis

Values are expressed as the mean ± SEM, and data were analyzed using one-way ANOVA with multiple comparisons to the control group at each time point post-EDS. For this purpose, GraphPad Prism (version 6, GraphPad Software Inc., San Diego, CA, USA) was used. Significant differences were regarded at *p* < 0.05, 0.01 or 0.001.

## 5. Conclusions

In summary, the present study demonstrates that MXC exposure during a specific window during development impaired Leydig cell differentiation and resulted in the delayed maturation of Leydig cell. The mechanisms of MXC disrupt Leydig cell development.

## Figures and Tables

**Table 1.** General parameters of toxicology after treatment of methoxychlor. EDS, ethane dimethane sulfonate.

Treatment	Control	Estradiol	Methoxychlor	

Dosage (mg/kg)	0	0.25	10	100
Body weight (g)				

14 days post-EDS	309.2 ± 4. 46	322.5 ± 4.88	360.2 ± 9.05 **	327.8 ± 2.24
32 days post-EDS	375.7 ± 5.75	354.3 ± 11.49	389.0 ± 9.09	380.7 ± 5.10
58 days post-EDS	424.3 ± 10.79	416.8 ± 19.01	413.5 ± 9.38	408.5 ± 6.97

Testis weight (g)				

14 days post-EDS	0.751 ± 0.008	0.779 ± 0.065	0.919 ± 0.132	0.875 ± 0.067
32 days post-EDS	0.896 ± 0.107	1.185 ± 0.133	1.186 ± 0.167	0.914 ± 0.149
58 days post-EDS	1.124 ± 0.099	1.448 ± 0.153	1.228 ± 0.119	1.136 ± 0.108

Mean ± SEM, *n* = 5.

** indicate significant difference compared to control group at *p* < 0.01.
